# Altered attention and visuospatial abnormalities in Parkinson's disease: links to attention networks and basal ganglia

**DOI:** 10.3389/fnagi.2026.1754795

**Published:** 2026-02-26

**Authors:** Jian Song, Yanyan Li, Yuqing Zhao, Haoping Gu, Jinfeng Xu, Xianling Xu, Wei Wei, Xiehua Xue

**Affiliations:** 1 The Affiliated Rehabilitation Hospital, Fujian University of Traditional Chinese Medicine, Fuzhou, China; 2 Fujian University of Traditional Chinese Medicine, Fuzhou, China; 3 Fujian Provincial Key Laboratory of Cognitive Rehabilitation, Fuzhou, China; 4 Fujian Provincial Key Laboratory of Rehabilitation Technology, Fuzhou, China

**Keywords:** amplitude of low-frequency fluctuations, attention function, functional connectivity, Parkinson's disease, resting-state functional magnetic resonance imaging

## Abstract

**Background:**

Attentional and visuospatial deficits in Parkinson's disease (PD) are linked to disrupted attention networks (AN), yet specific neural mechanisms remain unclear. This study aims to elucidate the relationship between these dysfunctions and aberrant AN circuits in PD.

**Methods:**

Sixty-two PD patients stratified by cognitive status [cognitively normal (PDCN), mild cognitive impairment (PDMCI), and dementia (PDD)] and 53 matched healthy controls (HC) underwent resting state functional MRI. We performed amplitude of low-frequency fluctuation (ALFF) and seed-based functional connectivity (FC) analyses. Relationships with cognitive functions were evaluated via partial correlation and mediation models. A support vector machine (SVM) classifier incorporating AN features was established for diagnostic validation.

**Results:**

Compared to HCs, PD patients showed increased ALFF in the right caudate nucleus (CAU) and left insular cortex (Ins), but reduced ALFF in the left middle occipital gyrus (MOG). FC analysis revealed decreased coupling in the right CAU-left cerebellum VI (CVI), left Ins-left middle cingulate gyrus (MCG), and left Ins-right superior temporal gyrus (STG), alongside enhanced left MOG-left inferior parietal lobule (IPL) connectivity. These alterations correlated significantly with cognitive performance. Mediation analysis identified left Ins ALFF and left Ins-left MCG FC as independent mediators of impairments. The SVM classifier achieved 92.2% accuracy (AUC = 0.944). Subgroup comparisons revealed increased ALFF in the left triangular inferior frontal gyrus (IFGtri) and angular gyrus (AG) in PDMCI vs. PDCN. PDD patients displayed decreased left supramarginal gyrus (SMG) ALFF compared to PDCN. Notably, left SMG-related alterations distinguished PDMCI from PDD and correlated with attention deficits.

**Conclusion:**

PD-related attention and visuospatial dysfunctions are closely linked to AN abnormalities. The Ins serves as a key regulatory node, while the SMG emerges as a potential neuroimaging marker for tracking cognitive decline from PDMCI to PDD.

## 1 Introduction

Parkinson's disease (PD) is characterized by cognitive impairment (Jones et al., 2021), the severity of which tends to increase as the disease advances. Approximately 80% of PD patients are at risk of developing PD with dementia (PDD) (Gallagher et al., 2021; Li R. et al., 2024). Attentional and visuospatial deficits are prominent features of cognitive impairment in PD (Hirano, 2021), manifesting as difficulties with sustained attention and impaired spatial orientation. These symptoms may emerge as early as the prodromal phase and are closely linked to an increased risk of falls and reduced functional independence (Pan et al., 2022a). Recent research has recommended the use of neuropsychological assessments targeting visuospatial function and related domains to better characterize the progression of cognitive impairment in PD patients, including PD with mild cognitive impairment (PDMCI) or PDD (Bezdicek et al., 2025). These assessments can facilitate patient stratification in clinical trials or serve as outcome measures. The heterogeneity of these clinical symptoms further suggests that the neural mechanisms underlying attentional and visuospatial deficits may vary across different stages of PD.

Attentional and visuospatial functions depend on the attentional network (AN), which encompasses key neural structures, whereas visuospatial processing is intricately linked to the posterior parietal-occipital visual integration network. Abnormal neural activity within the attentional and visual networks has been observed in PD patients with cognitive impairment. Studies have reported large-scale brain network dysfunction in PDMCI, including reduced functional connectivity (FC) in the cerebellar and AN (Shuai et al., 2020). Additionally, decreased cerebral perfusion in the posterior visual network (VN) and dorsal attention network (DAN) has been observed in cognitively impaired PD patients, indicating localized network dysfunction (Arslan et al., 2020). Furthermore, (Oh et al. 2020) demonstrated that reduced resting-state functional magnetic resonance imaging (rsfMRI) signal values in the right insular cortex (Ins) and right occipital cortex are associated with decreased dopamine transporter (DAT) uptake in the caudate nucleus (CAU), correlating strongly with deficits in executive and visuospatial functions. Collectively, these findings suggest that cognitive impairment in PD may stem from disrupted interactions between attentional and visual networks. However, it remains unclear whether these deficits are directly driven by aberrant neural activity specifically within core regions of the AN.

Moreover, research has identified stage-specific abnormalities in attention and visuospatial functions in PD. Studies suggest that cognitively normal PD patients (PDCN) may rely on cholinergic compensation to maintain relatively intact cognitive performance (Legault-Denis et al., 2024). However, in the PDMCI or PDD stages, more pronounced abnormalities in key hub regions or large-scale network disintegration (e.g., posterior cingulate cortex or intraparietal sulcus) become evident (Li X. et al., 2024). Nevertheless, current research lacks systematic comparisons across PDCN, PDMCI, and PDD subgroups, making it challenging to delineate the developmental trajectory of attention and visuospatial impairments across these subgroups. Furthermore, the specific contributions of core brain regions within the attention and visuospatial networks, such as the supramarginal gyrus (SMG) or posterior cingulate cortex, in PD-related attention and visuospatial deficits remain unclear.

To address these gaps, advanced neuroimaging techniques are essential. Previous rsfMRI studies investigating PD-related cognitive impairment have extensively utilized methods such as Independent Component Analysis (ICA) (Li X. et al., 2024), Graph Theory and Regional Homogeneity (ReHo) (Li et al., 2021). While ICA and Graph Theory offer valuable insights into global network topology and modular organization, they often lack the specificity to distinguish whether network failure originates from a primary lesion in a local node or from a disrupted information transfer along pathways. Similarly, while ReHo focuses on local synchronization, it may not fully capture the magnitude of regional activity.

To overcome these limitations and better characterize the pathophysiology, we employed a combined approach using Amplitude of Low-Frequency Fluctuations (ALFF) and seed-based FC. ALFF is a validated metric that reflects the intensity of spontaneous regional neural activity and local metabolic demands (Zang et al., 2007), whereas seed-based FC characterizes the temporal synchronization of blood oxygen level-dependent (BOLD) signals between spatially distinct regions (De Bartolo et al., 2025). By integrating these two metrics, our study offers a distinct methodological advantage: it allows us to distinguish local alterations in intrinsic activity (via ALFF) from disruptions in network synchrony (via FC). This specific combination enables us to test whether dysfunction in specific local nodes within the AN acts as a trigger that precipitates broader network-level disruption.

Based on this methodological framework, this study aims to answer two key scientific questions through the integration of ALFF and FC analyses: (1) Are attentional and visuospatial deficits in PD patients associated with abnormal neural activity in core regions of the attention network? (2) How does stage-specific reorganization of attention networks across different subgroups-namely PDCN, PDMCI, and PDD-influence attentional and visuospatial functions in PD patients? Clarifying these patterns may help identify neuroimaging biomarkers for earlier and more precise diagnosis and targeted interventions.

## 2 Materials and methods

### 2.1 Study participants

This cross-sectional study was conducted from March 2024 to May 2025 at the Rehabilitation Hospital affiliated with Fujian University of Traditional Chinese Medicine and surrounding communities (Fuzhou, China). We recruited 62 PD patients and 53 healthy control (HC) subjects matched for age, gender, and years of education. After receiving a comprehensive explanation of the research process, all participants voluntarily signed the written informed consent form. The study protocol was approved by the Ethics Committee of the Rehabilitation Hospital affiliated with Fujian University of Traditional Chinese Medicine (No. 2023KY-056-002).

### 2.2 Inclusion and exclusion criteria

Patients were included if they met the following criteria: (1) the PD cohort was diagnosed according to the Movement Disorder Society (MDS) Clinical Diagnostic Criteria for PD (Li et al., 2017); (2) subjects aged 18–80 years; right-handed; (3) subjects were required to voluntarily sign an informed consent form and be able to understand and cooperate with the assessment and MRI test of this trial; and (4) all PD patients underwent clinical evaluations and MRI acquisition while in the “ON” state.

Patients were excluded if they met the following criteria: (1) individuals exhibited parkinsonian syndromes or atypical parkinsonian disorders; (2) subjects presented with comorbid neurological conditions including cerebrovascular accidents or neoplastic lesions; and (3) patients demonstrated major psychiatric disorders, or significant audio-visual impairments who are unable to cooperate with evaluation or MRI testing.

Healthy controls were recruited to match the PD cohort in age, sex, and education. HC subjects were required to be free of major physical illnesses, history of PD, craniocerebral trauma, neurologic and psychiatric disorders and no contraindications to MRI scanning.

### 2.3 Clinical and neuropsychological evaluations

Cognitive function was assessed using the Montreal Cognitive Assessment (MoCA) scale. The PD cohort was stratified into three subgroups based on established criteria: PDCN subgroup (MoCA score ≥26), PDMCI subgroup (MoCA 19–25), and PDD subgroup (MoCA score ≤ 18) (Hoops et al., 2009). Consistent with the methodological framework described by (Lam et al. 2013), MoCA performance was further analyzed across five cognitive domains: (1) memory function; (2) visuospatial function; (3) language function; (4) attention function; and (5) executive function.

Given the aims of the present study, we focused primarily on the attention and visuospatial domain scores to characterize cognitive changes in the PD cohort and across subgroups.

### 2.4 Magnetic resonance imaging data acquisition

All subjects (PD patients and HC group) underwent MRI scans after completing the neuropsychological assessment. Magnetic resonance imaging was performed at the Rehabilitation Hospital Affiliated with Fujian University of Traditional Chinese Medicine. The scanning equipment uses a Siemens 3T Prisma MRI scanner and a standard head coil. Prior to scanning, participants were instructed to keep their eyes closed, remain awake, stay still, and avoid engaging in any specific thoughts. All participants wore earplugs to reduce scanner noise and were instructed to minimize head motion. The sequence and parameters of MRI scanning are as follows:

Structural images were acquired using a T1-weighted magnetization-prepared rapid gradient-echo (MPRAGE) sequence (sagittal): repetition time (TR) = 2,200 ms, echo time (TE) = 2.48 ms, the field of view (FOV) = 250 × 250 mm^2^, number of slices = 176, slice thickness = 1 mm, imaging matrix = 256 × 256.

Resting-state fMRI data were acquired using a gradient-echo echo-planar imaging (GE-EPI) sequence (axial): TR = 2,000 ms, TE = 30 ms, FOV = 224 × 224 mm^2^, number of slices = 37, slice thickness = 3.5 mm, imaging matrix = 64 × 64, and time points = 240.

### 2.5 Preprocessing and statistical analysis of resting-state functional magnetic resonance imaging

Functional image preprocessing was carried out using theMatrix Laboratory (MATLAB, MathWorks, USA, version 2018b) with Data Processing and Analysis for Brain Imaging (DPABI) software (Yan et al., 2016). For each participant, the first 10 volumes (time points) were discarded to allow for signal equilibration and participant adaptation. Subsequently, head motion parameters were calculated for all participants in this study, and those exceeding a threshold of 3 mm translation or 3° rotation on any axis were excluded. None of the participants exhibited excessive head motion. In addition, after an independent sample *t*-test, no significant differences were found between the head movement data of the two groups of subjects (*P* > 0.05).

Subsequently, nuisance covariates were regressed out, including the 24-parameter Friston motion model (Jia et al., 2020) and signals from white matter, and cerebrospinal fluid. Finally, T1-weighted images were utilized to align with the functional images. The Diffeomorphic Anatomical Registration Through Exponentiated Lie Algebra (DARTEL) method was used to normalize the fMRI image space to a standard Montreal Neurological Institute (MNI) template and resampled to a voxel size of 3 × 3 × 3 mm^3^.

### 2.6 ALFF analysis

We performed ALFF analysis using Data Processing Assistant for Resting-State fMRI (DPARSF) (Chao-Gan and Yu-Feng, 2010) (http://www.restfmri.net). For each voxel, the preprocessed time series was transformed into the frequency domain using a fast Fourier transform (FFT). The square root of the power spectrum was then calculated at each frequency, and ALFF was obtained by averaging amplitudes within the 0.01–0.10 Hz band to reduce physiological and scanner-related noise (e.g., respiratory and cardiac effects) and low-frequency drift (Jia et al., 2020). Afterward, the obtained ALFF values were standardized using *z*-score normalization. Finally, the standardized ALFF underwent smoothing using a 4 mm full width at half maximum (FWHM) Gaussian kernel.

### 2.7 FC analysis

We utilized DPARSF (Chao-Gan and Yu-Feng, 2010) (http://www.restfmri.net) for FC value calculations. Based on the between-group ALFF comparison, clusters showing significant differences were used as masks and defined as regions of interest (ROIs) for subsequent seed-based FC analysis. Temporal band-pass filtering (0.01–0.10 Hz) and linear detrending were applied to reduce low-frequency drift and high-frequency noise. The preprocessed data were spatially smoothed using a 4-mm FWHM Gaussian kernel. For each participant, Pearson's correlation coefficients were calculated between the mean time series of each ROI and the time series of all other voxels. Correlation coefficients were transformed to *z*-scores using Fisher's *r*-to-*z* transformation, yielding an individual FC map for each participant.

### 2.8 Construction of support vector machine model

To classify PD patients from HC subjects, we trained a support vector machine (SVM) with a Gaussian (radial basis function) kernel using R (v4.3.1). Model performance was assessed using accuracy, sensitivity, specificity, F1 score, and the area under the receiver operating characteristic curve (AUC). We used 10-fold cross-validation. Within each fold, 90% of the data were used for training and 10% for testing.

Given the significant group differences in ALFF values and FC values which were also significantly correlated with attention scores, we incorporated these variables into SVM models. Four distinct SVM models were constructed. Each model included basic covariates (age, sex, and years of education) and attention subscore, together with different neuroimaging features (ALFF and/or FC values).

### 2.9 Statistical analysis

General demographic and clinical data were analyzed by SPSS software version 26.0 (IBM Corporation, Armonk, NY, USA), where categorical information such as gender was tested using the chi-square test, and independent sample *t*-tests or Mann-Whitney *U*-test were used for age, educational years, and MoCA score and subscores.

For neuroimaging data, this study used independent sample *t*-tests in DPARSF to determine differences in ALFF and FC values between PD group and the HC group. Among PD subgroups (PDCN, PDMCI, and PDD), one-way analysis of variance (ANOVA) was used, followed by Bonferroni-corrected *post hoc* tests for pairwise comparisons. DPARSF was used to identify peak values within significant clusters. Multiple comparison corrections were applied using the Gaussian random field (GRF) correction (cluster-level threshold of *P* < 0.05 and a voxel-level threshold of *P* < 0.001, two-tailed). This study defined clusters for statistical analysis with a volume greater than 5 in the ALFF data analysis and clusters with a volume greater than 5 in the FC value data analysis.

This study conducted correlation analysis to explore the relationship between abnormal ALFF and FC values and clinical features. Partial correlation analyses were calculated to assess the relationship between spontaneous brain activity (ALFF and FC values) and clinical data, focusing on regions with statistically significant inter-group differences. The partial correlation analysis controlled for age, gender, and educational years. Finally, partial correlations of behavioral measures (e.g., the attention score) with ALFF and FC values in the PD group were analyzed.

In this study, variables that were significant in the partial correlation analysis were selected for mediated moderation model analysis. After controlling for participants' age, gender, and educational years, we used the PROCESS macro (V4.1) in SPSS V26.0 to estimate the possible mediating effects of significant ALFF and FC values on attention function. In the mediated moderation model, the independent variable was the group variable (PD group = 1, HC group = 2), the dependent variable was the attention score, and the mediators were ALFF and FC values. All mediated moderation model analyses were based on ordinary least squares regression with non-parametric bootstrap procedures (5,000 trials), which resulted in bias-corrected confidence intervals (CIs) for the effect size inference. *P* < 0.05 was considered statistically significant (a significant effect was indicated if the 95% CI did not cover the null).

## 3 Result

### 3.1 Clinical demographic characteristics

The study found that the PD group showed no statistically significant differences compared to HC group in terms of gender (*P* = 0.134), age (*P* = 0.075), and years of education (*P* = 0.107).

In contrast, the PD group exhibited significant differences in the total score of the MoCA as well as in the subscores for memory, visuospatial, attention, and executive (*P* < 0.05). However, there was no statistically significant difference in the language subscore between the PD group and HC group (*P* = 0.083), see Table 1.

**Table 1:** Demographic and clinical information of the participants.

**Item**	PD group (***n*** = 62)				**HC group (*n* = 53)**	***t*/*Z*/*x*^2^**	**Cohen's *d***	***P***
	**Total (*****n*** = **62)**	**PDCN (*****n*** = **26)**	**PDMCI (*****n*** = **24)**	**PDD (*****n*** = **12)**				
Gender (male/female)	34/28	14/12	15/9	5/7	21/32	2.651	–	0.134
Age (years)^#^	71.50 (65.75, 75.00)	70.00 (62.00, 74.00)	72.00 (65.50, 75.00)	70.00 (66.25, 77.50)	67.00 (65.00, 72.00)	−1.781	8.789	0.075
Years of education (years)^#^	12.00 (9.00, 12.00)	12.00 (8.75, 12.00)	12.00 (9.00, 12.00)	12.00 (9.00, 12.00)	10.00 (7.50, 12.00)	−1.615	2.826	0.107
Disease duration (years) ^#^	4.00 (2.50, 7.00)	5.50 (2.75, 7.25)	4.50 (2.63, 5.38)	2.75 (2.00, 4.50)	–	–	–	–
Hoehn-Yahr stage^#^	3.00 (2.00, 3.00)	3.00 (2.00, 3.00)	3.00 (3.00, 3.00)	3.00 (2.00, 3.00)	–	–	–	–
MDS-UPDRS III score	33.00 ± 13.81	29.88 ± 12.38	32.50 ± 14.37	40.75 ± 13.71	–	–	–	–
MoCA total score^#^	24.00 (21.00, 28.00)	28.00 (27.00, 29.00)	22.00 (21.00, 24.00)	14.00 (12.00, 17.75)	28.00 (28.00, 29.00)	−6.086	1.764	**< 0.001**
Memory subscore^#^	9.00 (7.00, 11.00)	11.00 (10.00, 11.00)	8.00 (7.00, 10.00)	4.00 (3.00, 6.00)	11.00 (10.00, 11.00)	−4.821	1.502	**< 0.001**
Visuospatial subscore^#^	3.00 (2.00, 4.00)	4.00 (3.75, 4.00)	3.00 (2.25, 3.75)	2.00 (1.25, 2.00)	4.00 (4.00, 4.00)	−5.369	0.639	**< 0.001**
Language subscore^#^	5.00 (4.00, 5.00)	5.00 (5.00, 5.00)	4.00 (4.00, 5.00)	3.00 (3.00, 4.00)	5.00 (4.00, 5.00)	−1.771	0.387	0.083
Attention subscore^#^	4.00 (3.00, 4.00)	4.00 (4.00, 4.00)	4.00 (3.00, 4.00)	2.00 (2.00, 3.00)	4.00 (4.00, 4.00)	−4.764	0.677	**< 0.001**
Executive subscore^#^	4.00 (2.00, 5.00)	5.00 (4.00, 5.00)	3.00 (2.00, 4.00)	2.00 (2.00, 2.75)	5.00 (4.00, 5.00)	−4.885	1.070	**< 0.001**

^#^If the data do not conform to the normal distribution, a non-parametric rank sum test is used, which is described by Median (P25, P75). The bolded values indicate *P* < 0.05.

### 3.2 Results of ALFF between the PD group and the HC group

**Figure 1:**
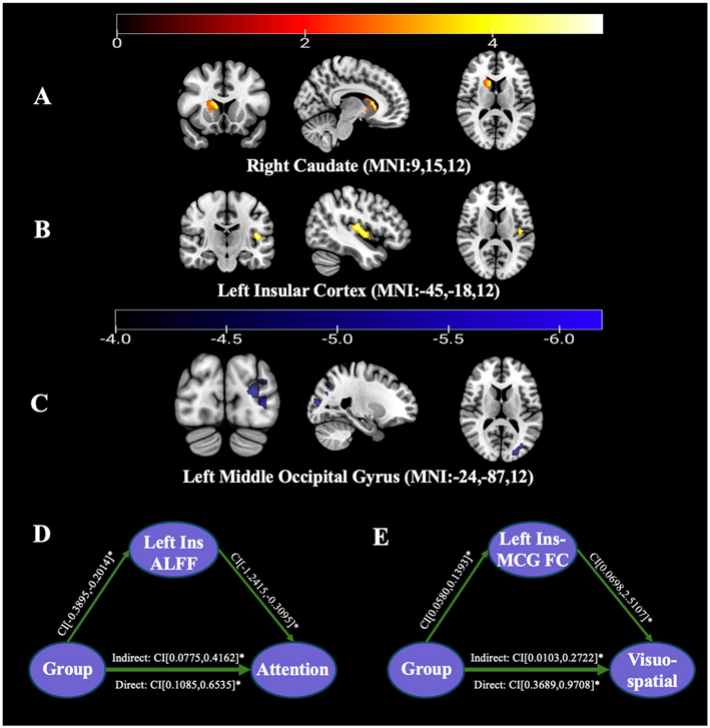
Results of ALFF values and mediated moderation model between the PD group and HC group. **(A)** ALFF map of the right CAU between the PD group and HC group (ALFF value increased). **(B)** ALFF map of the left Ins between the PD group and HC group (ALFF value increased). **(C)** ALFF map of the left MOG between the PD group and HC group (ALFF value decreased). **(D)** The mediated moderation model constructed with group as the independent variable (62 PD patients and 53 HC group), the attention subscore as the dependent variable, and the ALFF value of the left Ins as a mediator variable. **(E)** The mediated moderation model constructed with group as the independent variable (62 PD patients and 53 HC group), the visuospatial subscore as the dependent variable, and the FC value of left Ins-left MCG as a mediator variable.

Compared to the HC group, the PD group exhibited increased ALFF values in the right CAU and the left Ins, while a decrease in ALFF was observed in the left MOG. No other regions survived GRF correction and were therefore not included in further analyses, see Table 2, Figures 1A–C.

**Table 2:** The comparison of the inter-group differences in the amplitude of ALFF in different brain regions between the PD group and the HC group.

**Cluster**	Peak MNI coordinate			**Brain area**	***t***	**Voxels**
	***x***	***y***	***z***			
1	9	15	12	Right caudate nucleus (CAU)	7.134	192
2	−45	−18	12	Left insular cortex (Ins)	5.357	69
3	−24	−87	12	Left middle occipital gyrus (MOG)	−6.437	413

### 3.3 Results of FC between the PD group and the HC group

Compared with HC group, the FC value between the right CAU and left cerebellum VI (CVI) was decreased when the right CAU was used as ROI.

When the left Ins was used as the ROI, the FC values between the left Ins and both the left middle cingulate gyrus (MCG) and the right temporal pole of the superior temporal gyrus (STGtpo) were decreased.

**Figure 2:**
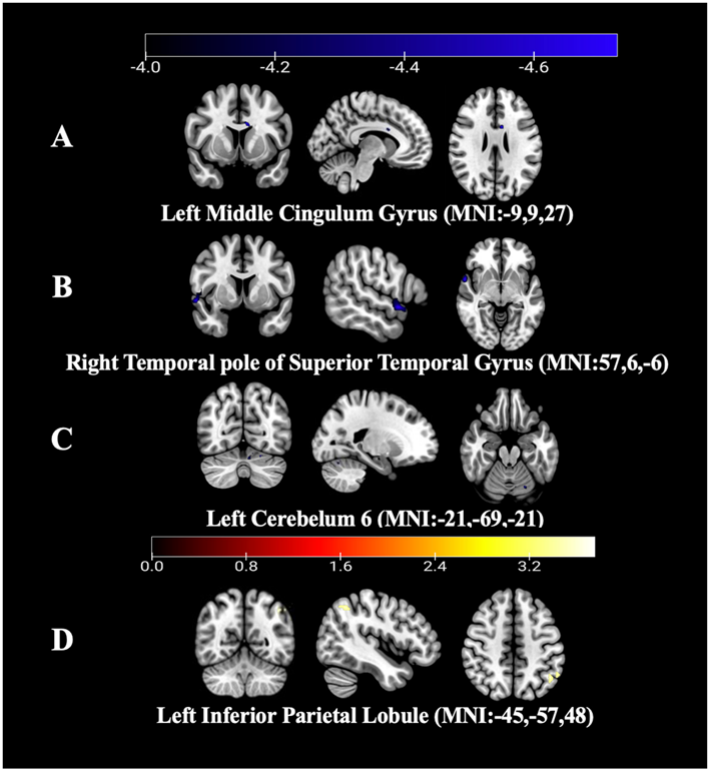
Results of FC values between the PD group and HC group. **(A)** FC map of the left Ins with the left MCG between the PD group and HC group (FC value decreased). **(B)** FC map of the left Ins with the left STGtpo between the PD group and HC group (FC value decreased). **(C)** FC map of the right CAU with the left CVI between the PD group and HC group (FC value decreased). **(D)** FC map of the left MOG with the left IPL between the PD group and HC group (FC value increased).

When the left Middle Occipital Gyrus (MOG) was used as ROI, FC value between the left MOG and the left inferior parietal lobule (IPL) was found to be increased. In the ROI-based FC analysis, no other brain regions were found to be involved through GRF correction, see Table 3, Figure 2.

**Table 3:** The comparison of inter-group differences in FC in different brain regions between the PD group and the HC group.

**ROI**	**Cluster**	Peak MNI coordinate			**Brain area**	***t***	**Voxels**
		***x***	***y***	***z***			
Right CAU	1	−21	−69	−21	Left cerebellum VI (CVI)	−4.529	64
Left Ins	1	−9	9	27	Left middle cingulate gyrus (MCG)	−4.870	72
	2	57	6	−6	Right temporal pole of superior temporal gyrus (STGtpo)	−5.740	125
Left MOG	1	−45	−57	48	Left inferior parietal lobule (IPL)	3.936	29

### 3.4 Results of correlation analysis in the PD groups

Partial correlation analysis showed that the ALFF values of the left Ins (*r* = −0.373, *P* = 0.004) and right CAU (*r* = −0.303, *P* = 0.020) were significantly negatively correlated with the attention subscore after controlling for age, gender, and years of education.

Partial correlation analysis showed that the ALFF value of the left MOG was significantly positively correlated with the attention subscore (*r* = 0.278, *P* = 0.033) and visuospatial subscore (*r* = 0.314, *P* = 0.015) after controlling for age, gender, and years of education.

FC-based partial correlation analysis (controlling for age, gender, and years of education) found that the FC value between the right CAU and the left cerebellum VI (*r* = 0.307, *P* = 0.018), the FC value between the left MOG and the left IPL (*r* = 0.259, *P* = 0.048), were significantly and positively correlated with the attention subscore.

**Figure 3:**
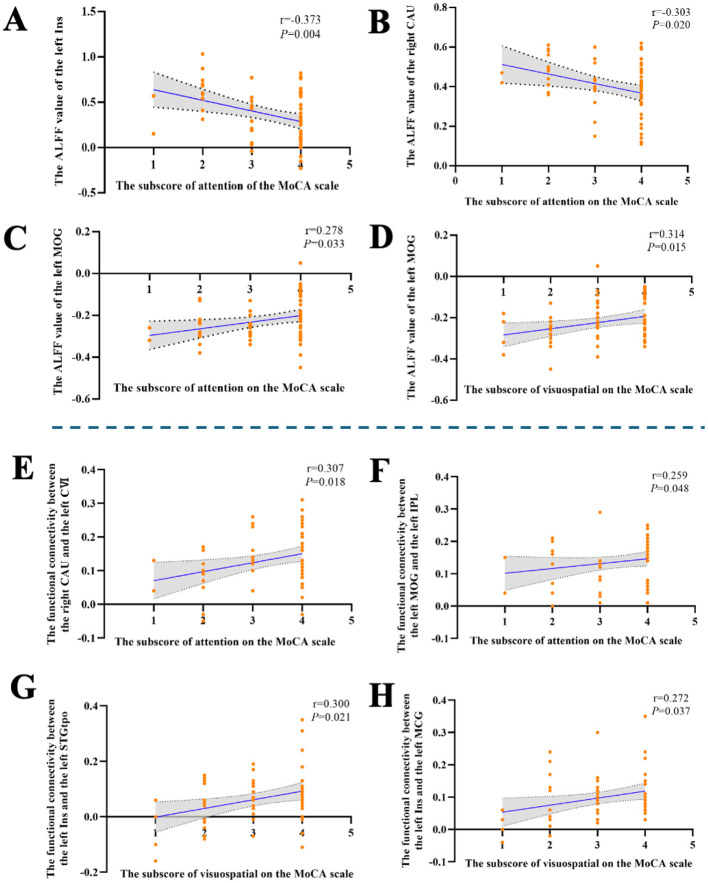
The results of the correlation analysis for the PD group. **(A)** Significant negative correlation between the ALFF value of left Ins and the attention subscore. **(B)** Significant negative correlation between the ALFF value of right CAU and the attention subscore. **(C)** Significant positive correlation between the ALFF value of left MOG and the attention subscore. **(D)** Significant positive correlation between the ALFF value of left MOG and the visuospatial subscore. **(E)** Significant positive correlation between the FC value of right CAU-left CVI and the attention subscore. **(F)** Significant positive correlation between the FC value of left MOG-left IPL and the attention subscore. **(G)** Significant positive correlation between the FC value of left Ins-left STGtpo and the visuospatial subscore. **(H)** Significant positive correlation between the FC value of left Ins-left MCG and the visuospatial subscore.

FC-based partial correlation analysis (controlling for age, gender, and years of education) found that the FC value between the left Ins and the left STGtpo (*r* = 0.300, *P* = 0.021), the FC value between the left Ins and the left MCG (*r* = 0.272, *P* = 0.037), were significantly and positively correlated with the visuospatial subscore, see Figure 3.

### 3.5 Results of the mediated moderation model analysis

In Model 1, a mediation-moderation analysis was conducted with group as the independent variable and attentional subscore as the dependent variable to examine the relationship between ALFF values in the left Ins and attentional performance.

In Model 2, group was again used as the independent variable, with visuospatial subscore as the dependent variable, to assess the FC value between the left Ins and the left MCG the relationship between FC values and visuospatial performance.

Both Model 1 and Model 2 demonstrated statistically significant differences. Specifically, the ALFF value in the left Ins (indirect 95% CI: [0.0775, 0.4162]) and the FC value between the left Ins and the left MCG (indirect 95% CI: [0.0103, 0.2772]) served as mediators for attentional and visuospatial subscores, respectively, see Figures 1D, E.

### 3.6 Results of ALFF among the PD subgroups

Compared to the PDCN group, the PDMCI group showed increased ALFF values in the left triangular part of the inferior frontal gyrus (IFGtri) and the left angular gyrus (AG).

Compared to the PDCN group, the PDD group exhibited decreased ALFF values in the left SMG and the left middle temporal gyrus (MTG), whereas the ALFF in the left IPL was increased.

**Figure 4:**
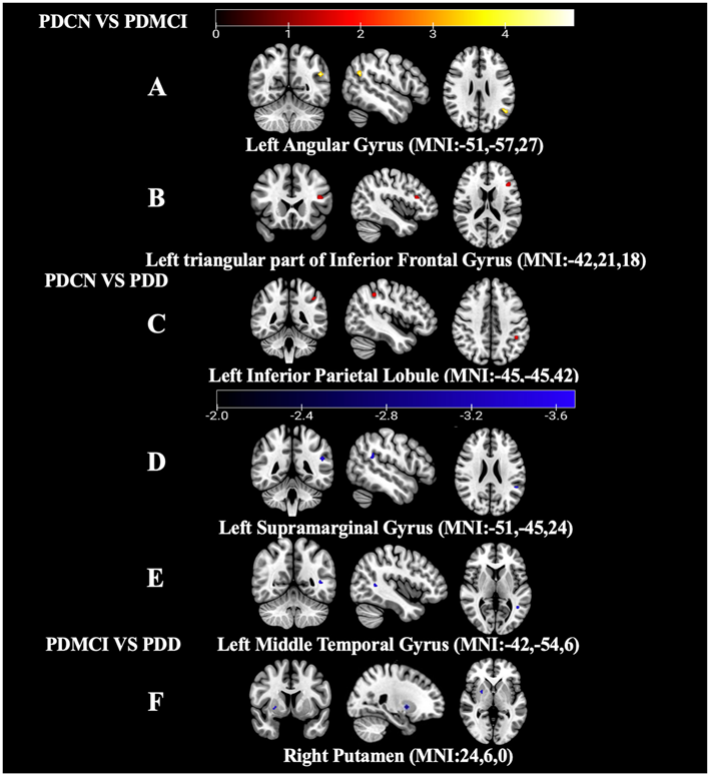
Results of ALFF values in the PD subgroups. **(A)** ALFF map of the left AG between the PDCN subgroup and PDMCI subgroup (ALFF value increased). **(B)** ALFF map of the left IFGtri between the PDCN subgroup and PDMCI subgroup (ALFF value increased). **(C)** ALFF map of the left IPL between the PDCN subgroup and PDD subgroup (ALFF value increased). **(D)** ALFF map of the left SMG between the PDCN subgroup and PDD subgroup (ALFF value decreased). **(E)** ALFF map of the left MTG between the PDCN subgroup and PDD subgroup (ALFF value decreased). **(F)** ALFF map of the right PUT between the PDMCI subgroup and PDD subgroup (ALFF value decreased).

Compared to the PDMCI group, the PDD group showed decreased ALFF value in the right putamen nucleus (PUT). Other brain regions did not survive GRF correction, and therefore were not included in the statistics, see Table 4, Figure 4.

**Table 4:** The comparison of inter-group differences in the amplitude of ALFF in different brain regions between the PD subgroups.

**Cluster**	Peak MNI coordinate			**Brain area**	***t***	**Voxels**
	***x***	***y***	***z***			
**PDCN vs. PDMCI**						
1	−51	−57	27	Left angular gyrus (AG)	5.1627	12
2	−42	21	18	Left triangular part of inferior frontal gyrus (IFGtri)	4.5620	12
**PDCN vs. PDD**						
1	−45	−45	42	Left inferior parietal lobule (IPL)	4.2129	8
2	−51	−45	24	Left supramarginal gyrus (SMG)	−3.9174	5
3	−42	−54	6	Left middle temporal gyrus (MTG)	−3.7467	5
**PDMCI vs. PDD**						
1	24	6	0	Right putamen nucleus (PUT)	−3.7432	7

### 3.7 Results of FC among the PD subgroups

**Figure 5:**
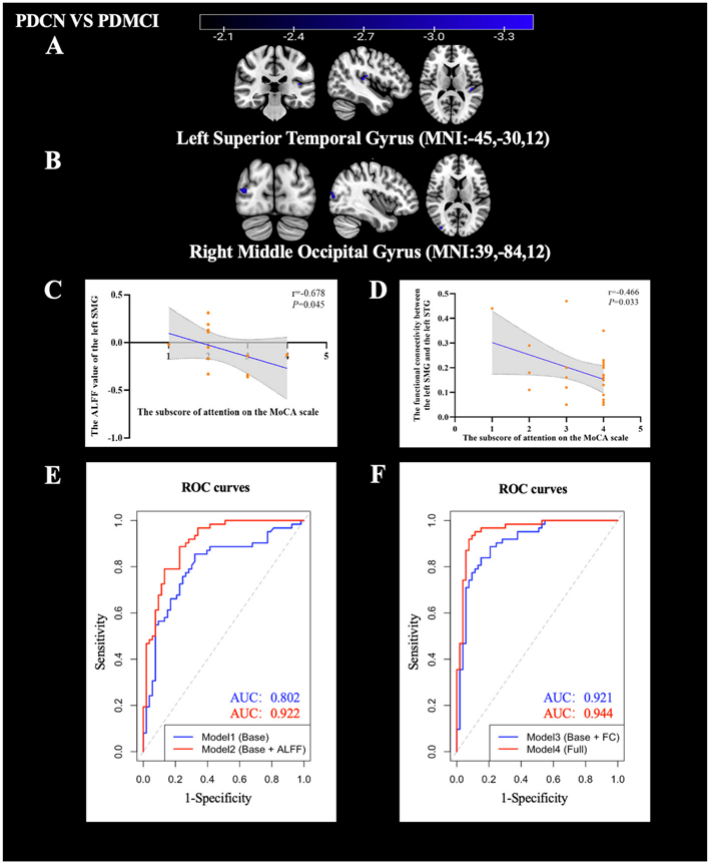
Results of FC values, correlation analysis and ROC analysis. **(A)** FC map of the left SMG with the left STG between the PDCN subgroup and PDMCI subgroup (FC value decreased). **(B)** FC map of the left SMG with the right MOG between the PDCN subgroup and PDMCI subgroup (FC value decreased). **(C)** Significant negative correlation between the ALFF value of left SMG and the attention subscore in PDD group. **(D)** Significant negative correlation between the FC value of left SMG-left STG and the attention subscore in PDMCI group. **(E)** The ROC curves results of the support vector models for Model 1 and Model 2. **(F)** The ROC curves results of the support vector models for Model 3 and Model 4.

Compared with PDCN, when the left SMG is used as the ROI, the FC values between the left SMG and the right MOG as well as the left STG was decreased in the PDMCI group. No significant FC differences were detected for the other ROIs, see Table 5, Figures 5A, B.

**Table 5:** The comparison of inter-group differences in FC in different brain regions between the PD subgroups.

**ROI**	**Cluster**	Peak MNI coordinate			**Brain area**	***t***	**Voxels**
		***x***	***y***	***z***			
Left SMG	1	−45	−30	12	Left superior temporal gyrus (STG)	−3.7175	5
	2	39	−84	12	Right middle occipital gyrus (MOG)	−4.0669	15

### 3.8 Results of correlation analysis in the PD subgroups

Partial correlation analysis showed that the ALFF value of the left SMG (*r* = −0.678, *P* = 0.045) was significantly negatively correlated with the attention subscore after controlling for age, gender, and years of education in the PDD group.

FC-based partial correlation analysis (controlling for age, gender, and years of education) found that the FC value between the left SMG and the left STG (*r* = −0.466, *P* = 0.033) was significantly and negatively correlated with the attention subscore in the PDMCI group, see Figures 5C, D.

### 3.9 ROC curves of SVM models in PD group and HC group

Model 1, which included the basic variables (sex, age, years of education, and attention subscore), achieved an accuracy of 73.30%, sensitivity of 79.70%, specificity of 67.90%, F1 score of 0.725, and an AUC of 0.802.

Model 2, which added ALFF values (right CAU, left Ins, and left MOG), achieved an accuracy of 82.80%, sensitivity of 78.30%, specificity of 86.90%, F1 score of 0.808, and an AUC of 0.922.

Model 3, which added FC values (right CAU–left CVI and left MOG–left IPL), achieved an accuracy of 83.40%, sensitivity of 77.00%, specificity of 89.00%, F1 score of 0.806, and an AUC of 0.921.

Model 4, which included both ALFF and FC values, achieved an accuracy of 92.20%, sensitivity of 89.00%, specificity of 95.00%, F1 score of 0.911, and an AUC of 0.944.

These findings indicated that the combination of ALFF values and FC values could provide better power for discriminating the PD group from the HC group, see Table 6 and Figures 5E, F.

**Table 6:** The results of the four SVM models.

**Item**	**Accuracy (%)**	**Sensitivity (%)**	**Specificity (%)**	**F1 score**	**AUC**
Model 1	73.30	79.70	67.90	0.725	0.802
Model 2	82.80	78.30	86.90	0.808	0.922
Model 3	83.40	77.00	89.00	0.806	0.921
Model 4	92.20	89.00	95.00	0.911	0.944

Model 1: basic variables included gender, age, years of education, and attention subscore. Model 2: included basic variables and the ALFF values (right CAU, left Ins, and left MOG). Model 3: included basic variables and the FC values (right CAU-left CVI and left MOG-left IPL). Model 4: included basic variables, ALFF values, and FC values.

## 4 Discussion

Compared to the HC group, the PD group exhibited increased ALFF values in the right caudate and left Ins (negatively correlated with attention) but decreased ALFF values in the left MOG (positively correlated with attention and visuospatial scores). These local alterations were accompanied by reduced FC values between the right caudate and left CVI, and between the left Ins and left MCG/right STGtpo, in contrast to increased left MOG–left IPL connectivity; notably, these aberrant FC patterns significantly correlated with attention or visuospatial metrics. Mediation modeling further identified left Ins ALFF and left Ins–MCG FC as specific mediators for attention and visuospatial functions, respectively. In subgroup analyses relative to PDCN, PDMCI patients showed increased ALFF in the left IFGtri and AG, whereas PDD patients displayed increased left IPL ALFF but decreased ALFF in the left MTG and left SMG. The latter negatively correlated with attention and showed reduced FC with the left STG (negatively correlated with attention) and right MOG. Additionally, the PDD group exhibited decreased right putamen ALFF compared to PDMCI patients. Furthermore, an SVM classifier utilizing these attention-related features achieved high diagnostic accuracy.

### 4.1 The changes in ALFF values in the PD group and HC group

Compared to the HC group, this study found that PD patients exhibited increased ALFF values in the right CAU, potentially indicating a compensatory mechanism in attention regulation. The CAU, a core component of the basal ganglia (Alves et al., 2022), is also integrated into the attention network and plays a critical role in modulating selective attention (Choi et al., 2023). Consistent with this, (Yoshimura et al. 2025) reported that reduced uptake of N-(3-[18F]fluoropropyl)-2β-carbomethoxy-3β-(4-iodophenyl) nortropane (FP-CIT) in the CAU of PD patients was associated with deficits in attention and working memory. Additionally, evidence suggests that involvement of the left CAU, a component of the brain's attention network, is crucial for processing speed in attention tasks (Botzung et al., 2019). Therefore, we speculate that in the PD cohort, dopaminergic neurodegeneration may trigger hyperactivation of the CAU as a compensatory mechanism to maintain attention allocation capabilities.

Furthermore, we observed elevated ALFF values in the left Ins of PD patients, suggesting that the Ins may serve as a mediating variable in attention function, potentially reflecting the recruitment of additional neural resources during attention tasks. The Ins is recognized for integrating sensory information and maintaining attentional orientation (Acunzo et al., 2025). Previous research has indicated that dysfunction in the left Ins may contribute to deficits in executive function and memory in early-stage PD patients (Pan et al., 2022b). Thus, the Ins, as a pivotal node in the attention network, may exhibit abnormal activation, which could be a key neural mechanism underlying attention deficits in PD patients.

This study revealed that PD patients exhibited reduced ALFF values in the left MOG, which positively correlated with scores of attention and visuospatial function. This reduction may exacerbate attention or visuospatial impairments in PD patients. The MOG, encompassing a significant portion of the visual cortex, has been shown to play a critical role in modulating category-selective attention (Liu et al., 2023) and is essential for visual information processing and spatial localization. Decreased ALFF values may reflect diminished functionality of the MOG in visual attention and spatial information integration, leading to difficulties in processing complex visual scenes or maintaining spatial orientation in PD patients. These findings suggest that functional abnormalities in the MOG may represent a potential neural mechanism underlying visuospatial and attention deficits in PD.

In summary, PD patients exhibited increased ALFF values in the right CAU and left Ins, alongside decreased ALFF values in the left MOG, revealing complex remodeling of the attention network and basal ganglia in PD. The observed abnormalities in attention and visuospatial functions in PD patients may be closely associated with aberrant activation in basal ganglia regions and the attention network.

### 4.2 The changes in FC values in the PD group and HC group

Compared with HC group, PD patients exhibited significantly reduced FC between the right CAU and the left cerebellum, which positively correlated with attention scores. This finding suggests abnormal neural network integration during attention-related tasks, potentially impairing the ability to sustain and shift attention. Previous studies have indicated that cerebellar dysfunction in PD patients contributes to deficits in attention control and social skills, a phenomenon referred to as “cerebellar cognitive affective syndrome” (Hirano, 2021). Additionally, research by (Kawabata et al. 2020) reported decreased FC between various cerebellar subregions and the basal ganglia in PD patients. These findings highlight that weakened FC between the CAU and cerebellum plays a critical role in attention deficits. We speculate that the left cerebellum is pivotal in attention processing, likely supporting the attention network through coordinated basal ganglia-cerebellum interactions. Furthermore, diminished FC may reflect disrupted basal ganglia–cerebellum coordination and thereby contribute to attention deficits in PD patients.

Additionally, reduced FC between the left Ins and both the left MCG and right STG was observed, with these reductions positively correlated with attention and visuospatial scores. Research by (Guo et al. 2025) demonstrated that in PD patients, a decline in the primary network gradient primarily occurs in the association cortex, encompassing the frontal cortex, Ins, cingulate cortex, and parietal cortex, corresponding to diminished function in the frontoparietal and ventral attention networks. Furthermore, studies have identified deficits in cholinergic activity in the superior temporal region of PD patients with hallucinations (d'Angremont et al., 2024). Additional research has shown that tracer uptake in brain regions such as the Ins, left dorsolateral prefrontal cortex, and posterior cingulate cortex is positively correlated with attention, executive function, and visuospatial abilities (d'Angremont et al., 2025). As a core node of the attention network, the Ins is critical for detecting salient stimuli and reallocating attentional resources. Reduced FC between the Ins and the MCG or STG may impair patients' ability to rapidly shift attention in dynamic environments and limit their capacity to efficiently allocate cognitive resources when processing complex information. The observed FC alterations involving the Ins suggest that cognitive impairment in PD is not merely a deficit in a single function but rather the result of synergistic abnormalities across multiple functional networks, including the frontoparietal, attention, and visual networks. Positron emission tomography (PET) studies further indicate that brain regions such as the cingulate cortex and Ins are most closely associated with attention and executive function, with cross-network implications (d'Angremont et al., 2025). Thus, the reduction in Ins -related FC may further suggest that abnormalities in the attention network indirectly impair attention and visuospatial performance in PD patients by disrupting information integration.

In contrast, FC between the left MOG and the left IPL is significantly increased in PD patients compared to HC group and positively correlates with attention scores. The IPL is a critical node in the attention network (Numssen et al., 2021), responsible for goal-directed attention allocation, while the MOG primarily processes visual information. Enhanced FC between these regions may reflect the brain's attempt to compensate for attention deficits by strengthening the interplay between visual and attention networks. This finding suggests that PD patients may rely on increased FC between specific brain regions to mitigate attention impairments, a pattern also observed in studies of MCI (Wu et al., 2023).

Taken together, these alterations in FC underscore the central role of the attention network in cognitive impairments associated with PD. They provide critical evidence for understanding the neural mechanisms underlying attention deficits in PD, suggesting that abnormalities in the attention network may influence attention and visuospatial functions through changes in FC.

### 4.3 The changes in ALFF values in the PD subgroup

Current evidence suggests that cognitive decline in PD is not merely a consequence of localized cortical dysfunction but originates from the disruption of integrated circuits, specifically the cortico-striato-thalamo-cortical (CSTC) loops (Paulo et al., 2023). Our findings support the “shared circuit” hypothesis, positing that the degeneration of dopaminergic nigrostriatal pathways triggers a cascading failure spanning from motor to associative loops.

Compared with the PDCN group, the PDMCI group exhibited significantly increased ALFF values in the left IFGtri, and the left AG. The left IFGtri, a key component of the prefrontal cortex (PFC), is critically involved in executive control and attention allocation. The left AG, located in the parietal lobe, is closely associated with attentional reallocation and multimodal information integration.

Previous studies have indicated that, as PD progresses, patients may increasingly rely on PFC-mediated neural substrates to compensate for attention and motor deficits (Assad et al., 2022). Furthermore, correlations between white matter integrity (fractional anisotropy) in the cingulate and parietal regions and AD-related biomarkers (Aβ-42/T-tau) underscore a link between these regions and overall disease burden (Assad et al., 2022; Huang et al., 2024). The increased ALFF in these two regions may reflect a compensatory mechanism in PDMCI patients, whereby heightened activity in the frontoparietal network supports the maintenance of attentional functions. This finding suggests that PDMCI patients may enhance localized neural activity in specific brain regions to counteract cognitive decline. Such a compensatory mechanism likely enables patients to sustain attention and cognitive flexibility during complex tasks, mitigating further cognitive deterioration. Moreover, this observation indicates early functional reorganization within the attention network in the PDMCI stage.

Compared with the PDCN group, the PDD group exhibited significantly reduced ALFF values in the left SMG and left MTG, with ALFF values in the left SMG showing a negative correlation with attention scores. Conversely, ALFF values in the left IPL were significantly increased. The left SMG, a critical node in the attention network, is implicated in goal-directed attention allocation. The observed reduction in its ALFF value may reflect pronounced deficits in attention maintenance and switching in PDD patients. (Grolez et al. 2020) reported that decreased activation in the right SMG—a key interface for information integration—as well as in the cerebellum and bilateral occipital cortex, is associated with cognitive slowing in PD patients. Furthermore, they noted that greater brain activation is linked to milder cognitive impairment. Another study identified the SMG/IPL as a critical region mediating goal-directed processing in visual attention, particularly in the context of working memory biases in humans (Wang et al., 2022).

The reduced ALFF value in the left MTG may further impair episodic memory or visuospatial information processing, potentially exacerbating attentional load indirectly, as patients require greater cognitive resources to process complex information. Research by (Yoo et al. 2024) demonstrated relatively decreased brain metabolism in the lateral temporal and frontal cortices projecting to the occipital and inferior parietal cortices. Alterations in visual perception may be associated with the ventral occipitotemporal pathway, broader temporal lobe function, and brain reserve in PD patients (Ohdake et al., 2020). The decreased ALFF value in the left MTG, coupled with associated functional abnormalities in the temporal-occipitotemporal pathway, significantly impacts episodic memory and visuospatial function in PD patients, indirectly increasing attentional load.

Notably, the observed MTG dysfunction potentially indicates a disconnection between the attention network and the medial temporal lobe memory system, supporting the “dual-hit” hypothesis involving both dopaminergic and cholinergic pathways (Reichmann, 2011). However, the elevated ALFF in the left IPL of PDD patients likely represents a late-stage compensatory effort to counteract SMG-related deficits.

Additionally, the elevated ALFF value in the left IPL in PDD patients may reflect a compensatory mechanism. As a pivotal region within the attention network, heightened IPL activity likely seeks to counteract attention deficits arising from impaired SMG function. A meta-analysis of neuroimaging studies indicates intrinsic functional abnormalities in the bilateral IPL and SMG in PD patients compared to HC group (Tahmasian et al., 2017). Additionally, research by (Kawashima et al. 2021) reported reduced IPL activation during working memory tasks in PD-MCI. Thus, this localized increase in IPL activity corroborates previous FC findings, suggesting that parietal regions may undergo functional reorganization to support residual attentional functions in PDD patients.

Compared to the PDMCI group, the PDD group exhibited significantly reduced ALFF value in the right PUT. This finding likely reflects further deterioration of basal ganglia function in the progression of cognitive impairment in PDD. The PUT, a critical structure within the basal ganglia, collaborates with the CAU in functions such as attention allocation and executive control. The observed decrease in ALFF value in the PUT may impair the basal ganglia-cortical circuits' support for the attention network, leading to further declines in attentional task performance in PDD patients. Research by (Esposito et al. 2021) found that dopamine depletion in the nigrostriatal pathway begins in the posterior PUT and is often more pronounced in PD. Certain visuospatial attention deficits observed in early PD may be linked to cognitive decline (Esposito et al., 2021). This perspective is corroborated by studies in dementia with Lewy bodies (DLB), where patients with visual hallucinations exhibit reduced gray matter volume in the right PUT, CAU, and Ins, which significantly correlates with visual attention performance (Pezzoli et al., 2019). Involvement of basal ganglia structures, particularly the left CAU and PUT, suggests their critical role in attentional processing speed in DLB patients (Botzung et al., 2019). Furthermore, PUT abnormalities may indirectly exacerbate dysfunction in the dorsal attention network by disrupting prefrontal-basal ganglia circuits, particularly during tasks requiring rapid attention switching or complex information processing. This diminished basal ganglia function may be associated with the more severe cognitive impairments in PDD, indicating that attention network abnormalities in advanced disease stages likely involve broader neural network damage.

Collectively, these findings elucidate the dynamic changes in the attention network across different stages of cognitive impairment in PD subgroups. These results underscore the pivotal role of the attention network in PD-related cognitive impairment, suggesting that its functional alterations, driven by localized neural activity abnormalities, significantly impact attentional performance and are closely associated with disease progression.

### 4.4 The changes in FC values in the PDMCI group

Compared to the PDCN group, PDMCI patients exhibited significantly reduced FC between the left SMG and the left STG, as well as the right MOG. These findings suggest that functional integration between the left SMG and these brain regions is impaired in PD, potentially exerting a significant impact on the attention network's functionality. The left SMG, a core node of the attention network, is critical for goal-directed attention allocation and spatial cognition. The reduced FC with the STG and MOG may compromise patients' performance in complex attentional tasks. Both physical frailty and cognitive impairment in PD have been associated with reduced gray matter volume in the lateral occipital cortex (Chen et al., 2019). Additionally, diminished connectivity in similar regions has been reported in PD patients with “wearing-off” effects, involving the temporal pole and inferior temporal gyrus (Zhai et al., 2024).

Research indicates that alterations in the parietal-occipital cortex of patients with early-stage Lewy body dementia may be associated with deficits in attention, executive function, and visuoperceptual/visuospatial abilities (Conti et al., 2024). These findings have been further corroborated in studies of MCI, where age-related changes in temporal lobe gray and white matter were more significantly correlated with performance on memory and attention tasks (Li et al., 2020). The STGtpo is involved in processing auditory-spatial information and multimodal integration, while the MOG primarily handles the initial processing of visual information. Reduced FC between these regions and the SMG may impair the integration of cross-modal information or the processing of visuospatial stimuli, leading to difficulties in sustaining and shifting attention. Such abnormalities in FC likely reflect the disruption of AN integration mechanisms due to the neurodegenerative pathology of PD, particularly in tasks requiring rapid allocation of cognitive resources.

Collectively, reduced FC values between the left SMG and both the left STG and right MOG highlight a core abnormality in the attention network of PD. The diminished FC between the SMG and STG, coupled with its negative correlation with attention scores, suggests that this connectivity impairment directly impacts attentional performance, likely by compromising the ability to integrate cross-modal information. These findings underscore the critical role of the attention network in PD-related cognitive impairment, with the SMG serving as a key node. Abnormalities in its FC may exacerbate attention deficits by disrupting the coordinated activity of neural networks.

This study has several limitations. First, the sample size was relatively small. In the current study, we performed an exploratory stratification of the PD cohort into three subgroups. Notably, the PDD subgroup comprised a relatively small sample size. Consequently, future studies with larger cohorts are warranted to further validate the consistency and robustness of these findings. However, we are actively recruiting additional PD patients and healthy participants to address this limitation in future studies. Second, this study was a cross-sectional analysis. Longitudinal studies with follow-up assessments of PD patients are needed to conduct more in-depth subgroup analyses and comprehensively evaluate changes in neuroimaging biomarkers during PD progression and across PD subgroups. Additionally, this study focused solely on functional MRI analysis. In the future, we plan to integrate multimodal MRI techniques to further enhance the significance of our findings.

## 5 Conclusion

Dysfunction within the attention network underlies attentional and visuospatial deficits in PD. The Ins emerges as a key regulator of attentional and visuospatial domains. Critically, subgroup analysis identifies the SMG—a core attention network node—as a potential predictive neuroimaging marker for progression from PD-MCI to PDD.

## Data Availability

Due to the restrictions on protecting participant privacy and ethical review, the original data of this study can be requested from the corresponding author upon reasonable request (Xiehua Xue, E-mail: f110015@fjtcm.edu.cn).
